# Exposure to distressing situations among registered nurses during the COVID-19 pandemic: a cross-sectional study

**DOI:** 10.1186/s12912-025-03249-9

**Published:** 2025-05-28

**Authors:** Dara Rasoal, Oili Dahl, Petter Gustavsson, Anna Ehrenberg, Ann Rudman

**Affiliations:** 1 School of Health and Welfare, Dalarna University, Högskolegatan 2, Falun, 79188 Sweden; 2 Division of Nursing, Department of Neurobiology, Care Sciences and Society, Karolinska Institutet. Function Perioperativ Medicin and Intensive Care, Karolinska University Hospital, Stockholm, Sweden., Stockholm, Sweden; 3 Division of Psychology, Department of Clinical Neuroscience, Karolinska Institutet, Stockholm, Sweden

**Keywords:** Registered nurses, COVID-19 pandemic, Intention to leave, Cross-sectional study, Distressing situations, Self-rated health, Job satisfaction

## Abstract

**Background:**

The COVID-19 pandemic exacerbated distressing situations among healthcare professionals, due to resource limitations and complex patient care challenges.

**Research objectives:**

The research aims to explore the frequency of RNs’ exposure to distressing situations across various care settings during the pandemic and assess the association between this exposure and: (1) stress of conscience, (2) intentions to leave the profession, (3) job satisfaction, and (4) self-rated health in the late phase of the pandemic. In addition, the aim was to describe other common situations they encountered during the pandemic.

**Research Design:**

A cross-sectional multi-method study was conducted with participants recruited from a national cohort of Registered Nurses (RNs) between October 2021 and January 2022. In total, 3,958 individuals met the eligibility criteria, with 2,237 participants (56.5%) responding to the survey. Among these respondents, 1,881 answered questions about distressing situations, and 239 shared open-ended responses about their experiences.

**Results:**

The results showed that during the peak of the COVID-19 pandemic, between 24% and 70% of RNs encountered distressing situations on a weekly basis or more often. Over 70% reported difficulties in communication due to personal protective equipment. Additionally, just over 40% of RNs reported working in situations lacking clear guidelines and facing prioritization challenges. RNs exposed to these distressing situations were frequently nearly twice as likely to experience stress of conscience (44% vs. 21%, [OR] = 2.87) and showed a stronger intention to leave the profession (25% vs. 14%, [OR] = 1.98). Moreover, they reported lower job satisfaction (85% vs. 92%, [OR] = 0.50) and poorer self-rated health (34% vs. 50%, [OR] = 0.52) compared to their counterparts with less exposure. In addition, RNs experienced a lack of support, understaffing, and working beyond their expertise, leading to emotional and physical exhaustion. They felt inadequate due to overwhelming workloads and limited recovery time.

**Conclusion:**

The COVID-19 pandemic has significantly impacted RNs, underscoring the need for strong organizational support and leadership. Nurses require guidance from leaders and institutions to manage distress and ethical challenges effectively. Future strategies should prioritize adequate staffing, skill development, teamwork, mental health resources, and transparent communication to support nurses’ wellbeing and recovery, ensuring the delivery of high-quality care.

## Background

The COVID-19 pandemic has had a profound impact on societies globally, affecting many aspects of daily life. Its effects have been especially felt across all sectors of healthcare, from primary healthcare to home care, nursing homes, and hospitals, all which have struggled with the challenges posed by the pandemic [1–4]. In a bid to control the spread of the virus, many countries implemented extraordinary measures, such as extensive closures of public spaces, schools, and businesses [5]. However, the Swedish government chose a different approach, keeping society relatively open instead of adopting the more common suppression strategies and full lockdowns. This unique strategy potentially resulted in certain outcomes, such as higher mortality rates among the elderly and increased strain on the healthcare system [6–8].

The COVID-19 pandemic subjected Registered Nurse (RNs) to diverse and challenging working conditions, including increased workloads, staff shortages, redeployments, and inadequate access to personal protective equipment, all of which placed significant strain on their capacity to provide care [9–12]. Additionally, these challenges were exacerbated by the emotional toll of continuously witnessing patient suffering and death [3, 13–16]. A notable issue arising under these conditions is being exposed to many distressing situations, which denotes the emotional turmoil RNs face when they are unable to act according to their ethical judgment due to various internal or external constraints [17–21]. Such distress is particularly prevalent among RNs navigating high-stakes or complex care scenarios [14].

In this study, we have defined *stress of conscience* as a comprehensive term for the general and moral distress and ethical and psychological stress experienced by registered nurses during the pandemic. Previous research has extensively explored the multifaceted nature of moral distress and its potential repercussions on both the professional and personal lives of RNs [14, 22]. Previous research has also described conscience as an ethical instinct, with stress of conscience being closely linked to ethical dilemmas or feelings of guilt that individuals impose on themselves when engaging in actions they perceive as morally wrong [23]. In the healthcare sector, particularly in nursing, staff often feel unable to provide the level of care they believe is necessary due to workplace constraints such as time pressure, conflicting demands, or resource limitations. This distress can lead to feelings of guilt, inadequacy, and emotional exhaustion [22, 24, 25]. This type of stress has been linked to emotional exhaustion and depersonalization among RNs [26]. It has also been linked to experiences of work intensification [27], which is a complex concept defined by the need to work more quickly, meet tighter deadlines, minimize idle time, and multitask.

However, there is a lack of studies that explore the extent of exposure RNs encountered to distressing situations across all areas of the healthcare system during the peak of the pandemic, and how this exposure may be associated with important outcomes such as stress of conscience, intention to leave the profession, job satisfaction, and self-rated health. This study is unique in its approach, as it includes a large sample of RNs that completed their nursing education in either 2002, 2004, or 2006. As a result, these RNs have similar post-graduation timeframes, yet they have acquired experience from a variety of care settings across the healthcare system. The research aims to explore the frequency of RNs’ exposure to distressing situations across various care settings during the pandemic and assess the relationship between this exposure and: (1) levels of stress of conscience, (2) intentions to leave the profession, (3) job satisfaction, and (4) self-rated health in the late phase of the pandemic. In addition, the aim was to describe other common situations they encountered during the pandemic. The insights gained from this research hold significant value as they have the potential to inform strategies aimed at enhancing the wellbeing and retention of RNs, ensuring they receive adequate support in future crises.

## Methods

This research employs a multi-method design to examine experiences of distressing situations among RNs during the COVID-19 pandemic. Quantitative data were collected through a survey to measure the frequency of exposure to distressing situations and their association with levels of stress of conscience, intention to leave the profession, job satisfaction, and self-rated health. The qualitative component involved open-ended survey responses describing frequently occurring situations at work during the pandemic.

### Design and participants

A cross-sectional multi-method design was utilized for this study. Study participants were part of a national cohort of RNs who responded to a survey between October 2021 and January 2022. The cohort comprises RNs longitudinally tracked since completing their nursing education in 2002, 2004, and 2006 [28]. Data collection was facilitated through surveys administered by the postal service. To enhance participation rates, two reminders were sent to respondents, and the survey could be completed either in printed form or online.

**Fig. 1:**
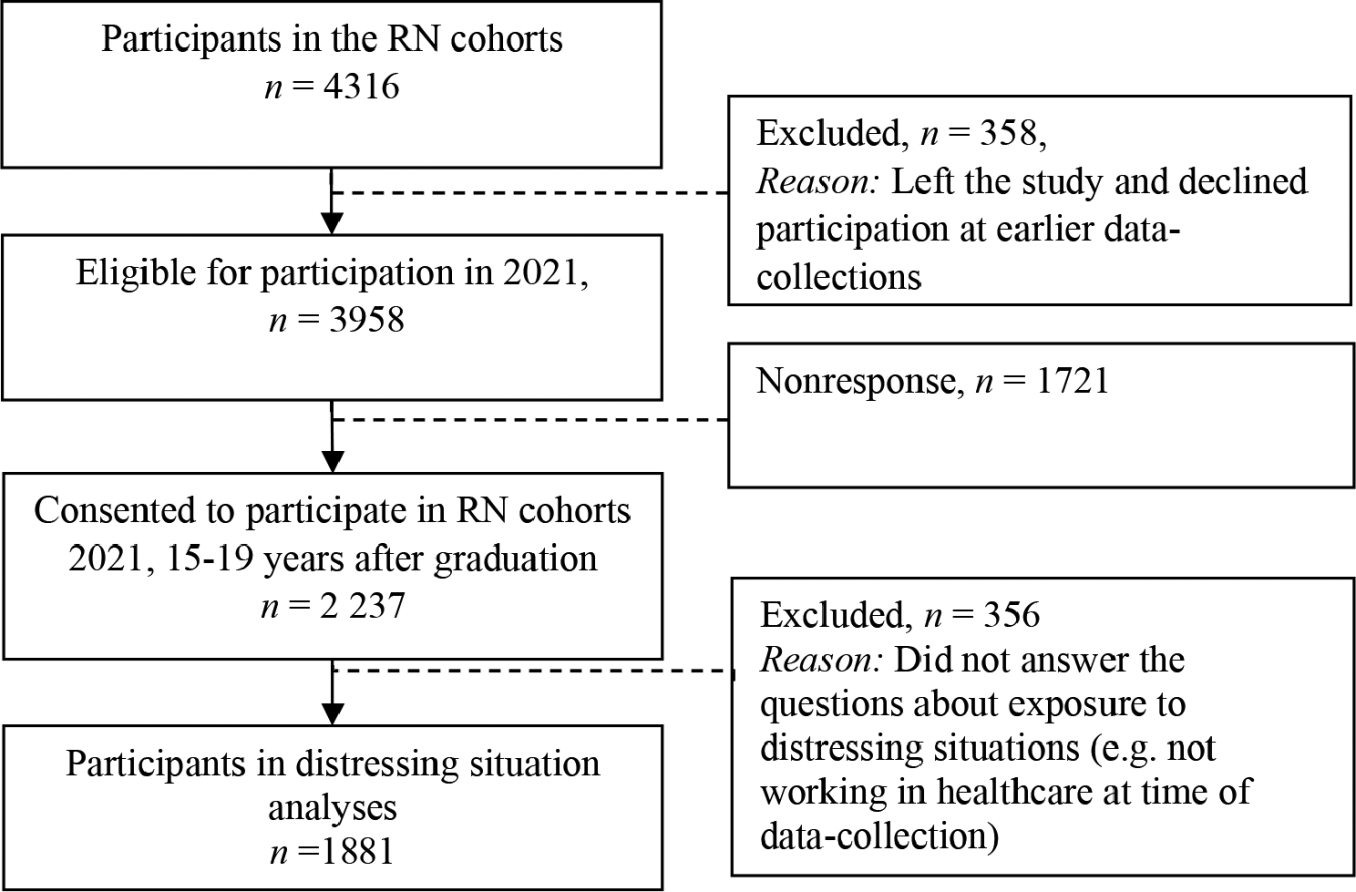
Flow chart illustrating the recruitment and participation process for the RN cohorts during the late COVID-19 pandemic 2021

For the 2021 data collection, 3,958 participants met the eligibility criteria, and of these, 2,237 individuals (56.5%) responded to the survey. Figure 1 displays the recruitment and participation process in the study during the late pandemic (i.e. the fourth quarter of 2021). It is worth noting that not all original participants were eligible for inclusion in 2021; some had declined further participation, relocated abroad, or were deceased (*n* = 358). The analytical sample used in this study consisted of RNs who responded to questions regarding distressing situations (*n* = 1,881). When the cohorts were initially established, their composition closely mirrored the student population within the nursing program, with minimal deviations– for more detailed information, see Rudman et al. 2010 [28]. Prior to the 2021 data collection, a remarkable 91.7% of the original cohort RNs remained available for participation after 15–19 years. To assess the representativeness of the available participants in 2021, we conducted an analysis based on various demographic factors. Respondents did not exhibit statistically significant differences between men (56.6%) or women (56.5%), or among participants with previously high (55.7%) or low (58.6%) levels of self-rated health. However, it is noteworthy that response rates varied significantly among age groups (based on birth year > 1970, 64.7%, 1971–1979, 54.1%, < 1980, 52.6%), with older participants showing a slightly higher response rate. In total, 239 participants responded to the open-ended question on common pandemic situations.

### Measurements

The exposure variable was the frequency of distressing situations encountered at work during the peak of the COVID-19 pandemic, and this was compared across various care settings. The outcome variables included stress of conscious, intention to leave the profession, job satisfaction, and self-rated health.

### Exposure to distressing situations

Exposure to distressing situations during the Covid-19 pandemic was assessed using nine items specifically developed for this study. These items were partly inspired by the Moral Distress Scale version 2 Kleinknecht-Dolf [29] and further developed by the research group in consultation with specialist RNs in older people care, surgery, intensive care, and prehospital care settings. Items 1–4 were chosen based on Kok et al.’s [30] description of prevalent sources of morally distressing work during the pandemic. Respondents were instructed to rate how often they had experienced the scenarios described in each item during the peak of the pandemic. The items and response scale were pilot tested by RNs from various different clinical specialties and healthcare contexts, resulting in the addition of the response option indicating an incidence of “every day”. Respondents were provided with the following options: (1) “Never”, (2) “Seldom”, (3) “Weekly”, (4) “Several times a week”, and (5) “Every day”. A mean value across all items was calculated representing the index of exposure to distressing situations for each respondent. This resulted in scale scores ranging from 1 (never) to 5 (every day). The Cronbach’s α coefficient for the scale was 0.873, which indicated high internal consistency. A cut off was established, dividing the responses into two groups: < weekly (comprising “Seldom” or “Never”) and > weekly (comprising “Every day”, “Several times a week”, and “Weekly”). This division reflects the extent to which RNs were exposed to distressing situations at work during the Covid-19 pandemic.

### Care settings

Care settings were categorised into three types: inpatient, outpatient, and home care.

### Stress of conscience

Respondents reported their level of stress of conscience through five items addressing their feelings of distress concerning delivering quality nursing care e.g. experiences of whether they could carry out nursing work at the level deemed necessary. The scale was inspired by the studies of Glasberg et al. [22] and Åhlin et al. [26]. The impact of conscience on nurses, particularly regarding feelings of guilt, is central to this concept. Nurses view conscience as a crucial element in their profession as it influences their actions with patients and their families, serving as a valuable guide in their pursuit of delivering high-quality care [31].

Examples of items used in this context included: “I often feel that it’s impossible to give patients, etc., the care they need,” “I am often forced to provide care that feels wrong,” and “I often have to compromise on my ambitions to provide good care”. Responses were given on a four-point scale ranging from 1 (applies completely) to 4 (does not apply at all). A mean value was calculated from the responses to all items and ranged from 1 to 4 (with 1 indicating low levels of moral distress and 4 indicating high levels of stress of conscience). The scale demonstrated good reliability, with a Cronbach’s α coefficient of 0.843. A cut-off was established dividing the responses into two groups: no stress of conscience (comprising Does not apply at all, Does not apply very well) and high stress of conscience (comprising Applies well, Applies completely).

### Intention to leave the profession

Respondents reported their intention to leave the profession through three items reflecting an overall turnover propensity. These items included statements such as “I think a lot about leaving the profession,” “I am actively looking for another job outside the nursing profession,” and “I will leave the nursing profession as soon as possible.” The scale was developed by Sjöberg and Sverke [32]. The items were rated using a 5-point scale ranging from 1 (strongly disagree) to 5 (strongly agree). The scale demonstrated good reliability with a Cronbach’s α coefficient of 0.828. A cut-off was established dividing the responses into two groups: low intentions to leave (comprising responses 1, 2, and 3) and strong intentions to leave (comprising responses 4 and 5, the two highest response alternatives).

### Job satisfaction

Respondents reported their level of job satisfaction through three items reflecting how often they felt satisfied with their work (i.e. I enjoy my work, I am satisfied with the job I have, I feel satisfaction with my work). The scale used was based on Brayfield’s attitude [33, 34] and was further developed by Sverke [32] as seen in [28] studies on RNs. The items were rated using a five-point scale, ranging from 1 (seldom or never) to 5 (very often or always). The scale demonstrates high reliability with a Cronbach’s α coefficient of 0.921. A cut-off was established by dividing the responses into two categories: low job satisfaction (comprising responses seldom or never, seldom, sometimes) and high job satisfaction (comprising responses often, very often, or always).

### Self-rated health

Respondents reported their overall health status by responding to a single item assessing their general state of health [35]. This item used a five-point response format ranging from 1 (poor) to 5 (good). A cut-off was established dividing the responses into two categories: poor health (comprising responses poor, pretty poor, neither good nor poor, and pretty good) and good health (comprising the response of good).

### Quantitative analysis

In the analyses, descriptive statistics were initially employed to estimate the prevalence of exposure to morally difficult situations. Subsequently, a descriptive analysis was conducted to investigate the association between exposure to distressing situations and care settings using χ^2^ tests, with the level of statistical significance set at *p* < 0.05 [36]. Post hoc tests were then performed to find out which care setting contributed most to the statistical association (i.e. adjusted standardized residuals with a z-value above 1.96 or under − 1.96). In the third step, individuals reporting high levels of exposure (i.e. > weekly) during the pandemic were classified as cases, while those reporting lower levels of exposure constituted the control group. Logistic regressions were utilized to estimate the odds ratio, comparing the odds of specific outcome measures (i.e. stress of conscience, intention to leave the profession, job satisfaction, and self-rated health) between the two groups, measured 21 months after the onset of the pandemic (OR crude). In the final step, information on where the RNs worked (i.e. care setting– inpatient, outpatient, or home health care) was added to the logistic regression model, and new odds ratios (OR adjusted) were calculated. Thus, care setting was included as a control variable in these logistic regression analyses. All analyses were conducted using SPSS statistics version 28.0 [37].

### Qualitative analysis

For the qualitative part of this study, an explorative design was employed, inspired by the research methodology for content analysis [38]. In total, 239 participants responded to the open-ended question on common pandemic situations. An open-ended question was included in the research survey to allow respondents to write down their experiences of being exposed to distressing situations during COVID-19. Specifically, respondents were prompted with questions like, “Please write down any common situations you have experienced during the pandemic.” This format provided the research participants with the possibility to express their thoughts and feelings in their own words, capturing the nuances of situations they encountered during the worst phase of the pandemic.

All documents containing participants’ responses to open-ended questions (*n* = 239) were organized into an Excel file. The first two researchers worked independently to read through and familiarize themselves with the content. Both authors (DR and OD) have clinical and academic experience, as well as training in qualitative methods. One researcher has more extensive clinical experience within the Swedish healthcare system, while the other approaches the topic from a primarily academic and analytical perspective. The researchers DR and OD reviewed all open-text responses multiple times to ensure thorough familiarization. An initial open coding process was then conducted by systematically tagging meaningful segments of text within the Excel sheets. Codes were generated inductively, allowing patterns to emerge directly from these open-text responses, and were subsequently organized into broader themes through an iterative process using a Word file. For example, codes such as “lack of managerial support” and “lack of skills, competence, and understaffing” were clustered under the theme “Uncertain Workplace Situation.” A thematic map was developed to visualize relationships between codes. This process was applied throughout the analysis of the entire dataset. Throughout the analysis, both researchers maintained a reflexive stance, remaining mindful of how their backgrounds, experiences, and assumptions could influence the interpretation of the data.

Themes were refined through discussions within the all the research group members and feedback to ensure coherence.

Any discrepancies were resolved through consensus, and some themes were reformulated for clarity. The first two authors discussed each theme with the last author (AR) to ensure it was well-defined and clearly differentiated. Findings were presented thematically, with illustrative quotes provided to support each theme. To enhance credibility, all authors were given the opportunity to comment on the final version of the results.

### Ethical considerations

This study was conducted in accordance with the ethical standards of the Declaration of Helsinki [39]. The study was approved by the Ethical Review Boards in in Stockholm, Sweden (Dnr 01–045; 04-587; 2016/793 − 32; 2021 − 00958; 2022-04898-02). Written informed consent was received from all respondents prior to them filling in the survey. The information provided to participants emphasized the voluntary nature of their participation, their right to withdraw at any time, and the assurance of confidentiality and secure data storage.

## Results

The study included a total of 1,881 RNs who completed the relevant questions for this study, and they were distributed across all 21 geographic regions in Sweden. Among the participants, 89% were female, with an average age of 48 years. Specifically, 11% were under 39 years old, 53% were between 40 and 49 years old, and 36% were over 50 years old. The majority, constituting 57% of respondents, had specialist training. Furthermore, 87% held clinical positions, while 6% held managerial roles. The most common care settings reported were outpatient facilities (41%), followed by inpatient units (37%), home health care (18%), and other workplaces (3%). The quantitative results will be presented first with references to tables, followed by the qualitative results, which will be presented through themes and subthemes and supported by quotes.

### Exposure to distressing situations

Table 1 presents the exposure of RNs to distressing situations during the peak of the COVID-19 pandemic, both by individual items and as an index. When collectively examining the prevalence of distressing situations faced by RNs 15–19 years after graduation during the pandemic’s peak, the results, as shown in the index in Table 1, showed that 69% encountered such situations on a weekly or more frequent basis. There was major variation in reported exposure to various types of situations, as presented by individual items in Table 1. The most commonly reported situation was difficulty in communicating through personal protective equipment, with just over 70% reporting experiencing this on a weekly or more frequent basis. The second most common situation reported was that relatives were not allowed to be with patients, with 65% of RNs experiencing this on a weekly or more frequent basis. The next most common situations, reported by slightly over 40% of RNs, included working in situations where clear guidelines were lacking, and the need to deprioritize essential work tasks on a weekly or more frequent basis. Less common situations, reported by 34% of the RNs, concerned issues related to fear of lacking the necessary competence or experience that was required for the task, as well as working with colleagues who may lack work skills. The frequency of exposure to distressing situations in various care settings during the peak of the COVID-19 pandemic is also shown in Table 1. RNs working in inpatient hospital care or home health care had significantly higher exposure to distressing situations than RNs working in outpatient settings.

**Table 1:** RNs’ exposure to distressing situations during the peak of the COVID-19 pandemic and the associations between care settings (inpatient/outpatient/home healthcare) (*n* = 1739). The number of participants ranges between 1858 and 1881 related to internal drop-out. Bold numbers indicate which cells in the contingency table contribute significantly to the Chi-square test (i.e. Adjusted standardized residuals with values > 1.96 or <-1.96)

	Exposure to distressing situations	> weekly*		< weekly**		> weekly Inpatient		> weekly Outpatient		> weekly Home care			
		*n*	%	*n*	%	*n*	%	*n*	%	*n*	%	χ^2^	*p*
1.	Was afraid of not having sufficient competence/experience for the work task	638	34.1	1235	65.9	**277**	**43.0**	**192**	**25.4**	123	36.7	49.0	0.001
2.	Worked with colleagues who did not have sufficient skills/experience to care for patients	641	34.2	1231	65.8	**310**	**48.3**	**142**	**18.8**	**140**	**41.7**	144.9	0.001
3.	Worked with colleagues who I think act in a way that is not safe for the patient	478	25.5	1394	74.5	**207**	**32.2**	**126**	**16.7**	**112**	**33.5**	57.1	0.001
4.	Provided suboptimal care due to insufficient resources, time, or personnel	636	34.2	1226	65.8	**266**	**41.4**	**177**	**23.6**	**133**	**40.1**	57.4	0.001
5	Did not have access to adequate protective equipment	533	28.4	1342	71.6	**206**	**32.1**	**178**	**23.5**	**110**	**32.8**	16.4	0.001
6	Difficulty in communicating through the protective equipment	1350	72.2	521	27.8	**517**	**80.4**	**479**	**63.4**	**267**	**80.4**	62.1	0.001
7	Relatives are not allowed to be with the patients	1202	64.7	656	35.3	**517**	**81.2**	**351**	**46.9**	**243**	**73.0**	189.7	0.001
8	Worked without clear guidelines	815	43.7	1049	56.3	**327**	**51.1**	**242**	**32.3**	**170**	**50.9**	60.9	0.001
9	Needed to prioritize away essential work tasks	767	41.2	1094	58.8	**303**	**47.3**	**255**	**33.9**	152	45.8	29.1	0.001
	Index of exposure to distressing situations	1299	69.1	582	30.9	**519**	**80.6**	**407**	**53.7**	**274**	**81.3**	141.4	0.001

Every day, Several times a week, Weekly. ** Seldom, Never

### Exposure to distressing situations and stress of conscience, intention to leave the profession, job satisfaction and self-rated health

Frequent exposure to distressing situations during the peak of the pandemic was associated with heightened stress of conscience, an increased likelihood of expressing a strong intention to leave the nursing profession, reduced job satisfaction, and poorer self-rated health during the late pandemic period (see Table 2). Specifically, RNs frequently exposed to distressing situations at the peak of the pandemic were nearly twice as likely to report higher levels of stress of conscience at work during the late pandemic period (44% vs. 21%, [OR] = 2.87; 95% [CI]: 2.28–3.61) and twice as likely to report strong intentions to leave the profession compared to their counterparts with lower exposure levels (25% vs. 14%, [OR] = 1.98; 95% [CI]: 1.52–2.58). RNs exposed to frequent distressing situations reported lower job satisfaction (85% vs. 92%, [OR] = 0.50; 95% [CI]: 0.36–0.70) and poorer self-rated health (34% vs. 50%, [OR] = 0.52; 95% [CI]: 0.42–0.63) compared to the RNs with lower levels of exposure. To account for potential variations based on where RNs worked, care settings were included in the regression equations. However, even after controlling for care setting, the associations remained about the same in magnitude (see Table 2). Thus, RNs exposed to distressing situations still reported higher stress of conscience at work during the late pandemic period, a stronger intention to leave, lower job satisfaction, and poorer self-rated health compared to RNs who had low levels of exposure.

**Table 2:** Prevalence and odds ratios of exposure to morally difficult situations (index > weekly vs. < weekly) and moral distress, intention to leave the profession, job satisfaction and self-rated health. Point estimates and confidence intervals are taken from a logistic regression analysis. The number of participants ranged between 1723 and 1868

	Estimates in the total sample			Logistic regression				
	Cohort	Exposed (> weekly)	Not exposed (< weekly)					
	*n* (%)	*n* (%)	*n* (%)	OR crude	95% CI	OR adjusted	95% CI	*p*
Moral distress	684 (36.9)	562 (43.8)	122 (21.4)	2.87	2.28–3.61	2.94	2.30–3.76	0.001
Intention to leave profession	398 (21.4)	316 (24.7)	82 (14.2)	1.98	1.52–2.58	2.16	1.62–2.89	0.001
Job satisfaction	1627 (87.2)	1098 (85.1)	529 (92.0)	0.50	0.36–0.70	0.51	0.35–0.73	0.001
Self-rated health	726 (38.9)	439 (34.0)	287 (49.8)	0.52	0.42–0.63	0.52	0.42–0.63	0.001

% Exposed to morally difficult situations = prevalence in the sample with exposure to morally difficult situations based on the index. OR = Odds ratio. % not Exposed to morally difficult situations = prevalence in the sample without exposure to morally difficult situations based on the Morally difficult situations index. OR crude = without statistically controlling for care settings. OR adjusted = statistically controlled for care settings

### Common situations experienced by nurses during the pandemic; open-ended responses

This qualitative analysis explores registered nurses’ (RNs) experiences during the COVID-19 pandemic, highlighting key challenges in their professional and personal lives. Thematic analysis identified two main themes: “Uncertain Workplace Situation” and “Unfulfilled Needs.” The first theme concerned challenges such as lack of managerial support, and lack of skills, competence, and understaffing, which contributed to workplace uncertainty. The second theme reflects the emotional toll on nurses, including difficulties in providing nursing care and a lack of recovery from work-related stress.

### **Theme 1.** Uncertain workplace situation

This theme included two subthemes that describe a range of common distressing situations RNs encountered in their work environment during the pandemic that induced a sense of uncertainty. The common types of distressing situations described were *Lack of managerial support*, with communication barriers being the most prominent, along with *Lack of skills*,* competence*,* and understaffing* that elicited uncertainty at work.

### Lack of managerial support

The lack of managerial support significantly worsened already challenging work conditions for RNs, contributing to heightened stress and a sense of vulnerability. Poor communication, unclear instructions, and inconsistent guidance from leadership left many RNs feeling isolated and unsure about how to navigate complex situations during the pandemic. The absence of effective managerial support meant that RNs often felt overwhelmed, with many reporting that their concerns and feedback were either ignored or dismissed.

“Our managers fled when COVID-19 arrived and worked from home. We had to handle many situations on our own and felt abandoned.” (RN A).

Poor communication from management during the pandemic not only created confusion but also left healthcare workers feeling unsupported and uncertain about how to handle rapidly changing guidelines and protocols.

“Initially, the information was inadequate and unclear. There was a lack of understanding and communication from management.” (RN B).

### Lack of skills, competence, and understaffing

The qualitative data also revealed that the COVID-19 pandemic had a profound impact on care settings, resulting in extreme workloads for RNs due to understaffing, resource shortages, and the redeployment of staff to roles outside their areas of expertise, which became common practice during the pandemic. These conditions exacerbated feelings of uncertainty and insecurity, as nurses were often required to perform tasks beyond their skills. The increased uncertainty not only diminished their professional confidence but also amplified their levels of stress and anxiety in an already challenging work environment. These combined pressures hindered their ability to provide optimal care, further intensifying the emotional and mental strain they experienced throughout the pandemic.

“We had to start working in the ICU without proper training. I was assigned to work as a nursing assistant instead of a registered nurse, with limited supervision.” (RN C).

This highlights the lack of proper training and the redeployment of staff to roles outside their expertise, contributing to feelings of uncertainty. RNs were often left to navigate complex and high-pressure situations without the necessary skills or experience, which increased their stress and anxiety.

“A new patient group, a new disease, and new colleagues in a constantly changing work environment made my job extremely stressful.” (RN D).

### **Theme 2.** Unfulfilled needs

The other common situations RNs faced during the pandemic extended beyond their professional duties, deeply affecting both their ability to provide care and their personal well-being. Two subthemes emerged from the analysis: *Lack of Nursing Care* and *Lack of Recovery*.

### Lack of nursing care

RNs experienced a heavy emotional toll during the pandemic, grappling with the inability to provide the level of care they aimed to offer, or, at times, providing suboptimal care due to overwhelming circumstances. This emotional burden was compounded by witnessing the suffering of patients who received delayed or inadequate treatment as healthcare systems struggled to meet demand. RNs not only felt the weight of their professional responsibilities but were also deeply affected by the emotional stress experienced by patients’ families, who were often unable to visit their loved ones due to restrictions.

“A sense of inadequacy, as care and nursing sometimes suffered because we were forced to prioritize among the most critically ill due to staff shortages and a heavy workload.” (RN E).

This illustrates the emotional burden and frustration RNs faced when they were unable to provide adequate care due to overwhelming circumstances. The inability to meet patients’ needs left many RNs feeling helpless and guilty, as they were forced to make difficult decisions about prioritizing care. Another RN expressed frustration:

“We were not able to provide patients with the care they needed.” (RN F).

Furthermore, caring for double the normal number of patients placed an immense strain on RNs’ ability to provide quality care. The overwhelming patient load often resulted in rushed or incomplete care, forcing nurses to make difficult compromises in their caregiving.

“We handled twice as many patients as usual and had to make tough prioritizations based on medical needs and assessments, which led to shortcomings in nursing care.” (RN G).

### Lack of recovery

This subtheme highlights how the pressures of work were compounded by challenges in RNs’ private lives during the pandemic. They had to balance minimizing the risk of infection with managing increased household responsibilities, all while facing a lack of recovery time. This lack of rest further deteriorated their mental health and overall wellbeing.

RNs in this study struggled to meet their basic needs, such as finding time to eat and sleep due to their demanding work shifts. The constant pressure of long working hours and overwhelming patient loads left them physically and emotionally exhausted, with no chance to recuperate between shifts, as one RN expressed:

“A feeling of inadequacy, difficulty in finding time to eat or use the restroom, and a severe lack of sleep. Long shifts with no recovery time and being reassigned.”

This inability to recover not only affected their personal well-being but also hindered their capacity to provide consistent, high-quality care, adding to their emotional and physical strain.

## Discussion

The research aimed to investigate RNs’ exposure to distressing situations at work during the COVID-19 pandemic. It also sought to explore whether this exposure was associated with stress of conscience, the intention to leave the profession, job satisfaction, and self-rated health among RNs. Additionally, the study aimed to describe other common situations that RNs encountered during the pandemic. Increased work intensification, unfamiliar work environments, and a lack of experience with the new disease exposed nurses to distressing situations, adding to the complexity of their professional responsibilities. Several studies describe nurses’ stress and its consequences during the pandemic that align with our findings [40, 41]. However, different expressions such as moral distress or psychological distress have been used interchangeably for the phenomenon of distress [40–42].

In the present study, 69% of registered nurses reported encountering distressing situations on a weekly basis or more frequently. Of those exposed, almost 50% reported high stress of conscience. Comparing these levels with other studies can be difficult because distress is defined in several ways and assessed using varying instruments and scales. However, distress reported at a high level during the pandemic by RNs in critical care is well recognised [40]. While numerous previous studies focus on RNs’ experiences of distress during the pandemic in critical care settings [35, 40], our study found that experienced RNs were subjected to significantly higher levels of stress of conscience, both in inpatient care and in residential care facilities. Additionally, Laher et al. reported a significant level of moral distress among staff working in nursing homes [43].

It is noteworthy that the included RNs in our study were all experienced nurses who had graduated 15 to 19 years ago. We initially believed that more work experience would serve as a protective factor for RNs against experiences of distress, however, another perspective suggests the opposite: that with more work experience, RNs may become more susceptible to distress, as they become more aware of the various consequences and risks affecting patient quality of care and safety. According to previous studies [40, 44], RNs with experience tend to have higher levels of moral sensitivity, which could make them more susceptible to distress. It is reasonable to expect that nursing experience influences adherence to ethical standards.

The factors affecting psychological distress among RNs during the pandemic have been classified into three main categories highlighting personal, care-related, and organizational factors [40–42]. Similar patterns emerged in our qualitative analysis, with nurses expressing personal constraints through feelings of helplessness and guilt over their patients’ unmet needs. Nurses often experience strong emotions when facing challenges in meeting patients’ needs, especially when required to make difficult decisions about care priorities. Burton et al. (2024) reported similar findings, with nurses describing experiences of inadequate care as ethical violations in care. Although nurses are trained to provide holistic patient care, the pandemic forced nurses to make challenging ethical decisions that often conflicted with their training and understanding [42].

Caring-related factors, as reported by nurses in our study, included a lack of teamwork skills and the experience necessary to handle complex and high-pressure situations. Heavy workloads and inadequate staffing exacerbated these stressors. Additionally, insufficient resources—such as medical supplies, available beds, and skilled staff—hindered nurses’ ability to provide optimal patient care [45].

The pandemic has significantly affected frontline nurses, requiring nurse managers to improve knowledge and skills, foster teamwork, and provide emotional support and recovery for staff [41]. In our study, nurses reported a lack of organizational support due to poor leadership and unclear communication regarding changing guidelines, resulting in feelings of abandonment. Inadequate leadership support has been recognized as a major organizational factor contributing to nurses’ distress during the pandemic [41, 42], often described as institutional betrayal due to insufficient resources. It involves a sense of being neglected, with insufficient access to both human and material resources [42]. Furthermore, nurses’ overwhelming workload during the pandemic is a potent cause of distress due to excessive double shifts, incoherent practices, and lack of recovery [42, 46]. Such conditions can result in both physical and mental exhaustion, which ultimately impacts the quality of care provided (38). Similarly, in our study, nurses noted that inadequate recovery time affected their well-being and ability to provide high-quality care, contributing to psychological and emotional exhaustion.

Furthermore, this study showed that RNs exposed to distressing situations were almost twice as likely to express intentions to leave the profession compared to those with no exposure. This finding aligns with other studies that have described a correlation between psychologically difficult situations and the intention to leave the profession [47]. There are several reasons for distressing situations during the pandemic. Increased distress due to an unsafe work environment, poor patient quality, and safety concerns have been identified as important predictors of nurses’ intention to leave the profession [15]. Additionally, the type of care setting affected the level of distress experienced; Registered Nurses (RNs) in outpatient care faced significantly fewer distressing situations during the pandemic. This finding adds new knowledge, as most studies on distress have primarily focused on inpatient settings [40].

Another interesting aspect of the findings in this study, as reported by the participating RNs, concerns difficulties in working with colleagues who lack the necessary skills for the job, as this could lead to compromised and unsafe patient care. Other sources of distress included insufficient resources in terms of both time and personnel. Previous studies have shown that various forms of resource scarcity can lead to unnecessary patient suffering, create a suboptimal work environment, and contribute to feelings of insecurity in providing care [13, 15, 18]. Three out of four registered nurses (RNs) reported weekly communication barriers due to protective equipment. Poor communication and team dynamics among RNs can negatively impact their mental health, increase stress, and compromise patient care [13, 18, 48].

Furthermore, the RNs in this study who were exposed to distressing situations reported significantly lower job satisfaction and poorer self-rated health compared to their counterparts without exposure. Previous studies indicate that RNs exposed to distressing situations tend to have lower job satisfaction and self-rated health [15], however, there are some critical nuances regarding these connections. It is difficult to ascertain whether distress causes lower job satisfaction and self-rated health or vice versa. It is entirely plausible that RNs with poorer health and lower job satisfaction might develop feelings of distress. Our study aligns with previous research findings indicating that RNs exposed to distressing situations have reported poorer health. Specifically, RNs in earlier COVID-19 studies have reported symptoms such as depression, sleep disturbances, anxiety, emotional instability, and exhaustion [47].

The findings from this study highlight the profound impact of the COVID-19 pandemic on RNs’ work. These data are important to recognize and understand long after the pandemic itself, partly because the long-term effects of exposure to distressing situations on the health and professional development of registered nurses remain uncertain. One possible consequence, however, could be a decline in job satisfaction and an increased intention to leave the profession. This may indicate that the healthcare sector could face significant challenges in retaining RNs in the future [49]. Additionally, a possible implication of distress could impact RNs’ engagement in providing high-quality care for patients. For society and the healthcare system as a whole, this could result in a crisis of confidence if the healthcare needs of the population cannot be met. It can also reduce capacity and resilience in the event of future challenges such as the pandemic or other serious events with limited healthcare resources, unless organizational changes are implemented to prevent distress.

To better support nurses during such situations, healthcare organizations and management should focus on several key areas:

- Ensure that nurses have the necessary protective equipment and medical resources to perform their work safely and effectively.
- Provide regular psychological support and counselling to help manage stress, anxiety, and burnout.
- Maintain open and transparent communication from management regarding changes, guidelines, and expectations.

This fosters a more supportive environment for nurses, helping them to thrive in difficult circumstances. Implement flexible work schedules to prevent burnout and enhance the work environment. In addition, continuous education and training are necessary to ensure that nurses are prepared to handle new challenges and tasks that may arise during serious extraordinary events [49]. Although the findings presented in this study are based on experiences during the COVID-19 pandemic, they offer important lessons that can inform preparedness planning and organizational strategies for future public health emergencies.

### Strengths and limitations

In terms of demographics, the respondents in this study accurately represented the entire population of nursing students across three national cohorts when recruited, and the long-term follow-up had a response rate of almost 60% in Sweden, which is significantly higher than what is typically observed. Thus, a major strength of this study is that it explores distress of RNs from a variety of workplaces and care contexts, where different types of care are provided to patients, rather than focusing solely on critical care settings, and gives space for nurses’ own stories about distressing situations. However, it is important to consider that all data collected in this study relied on self-reported information, which may introduce bias due to socially desirable responses [50]. Nevertheless, self-reported data are valuable for collecting information from diverse samples, especially when the phenomenon, such as experiencing a critical incident, cannot be directly observed or ethically recreated. The substantial number of RNs who reported exposure to distressing situations indicates that they were being open about their experiences, thus minimizing the influence of social desirability bias. As described in the methods section, a nine-item scale was developed to assess individuals’ exposure to distressing situations during the COVID-19 pandemic. This scale was specifically developed to fit the demanding work environment of the pandemic and was further refined after input from expert RNs specializing in care of older adults, surgery, intensive care, and prehospital care. To ensure the suitability of the items and response options, RNs from various specialties and care contexts participated in pilot testing, which led to the inclusion of a response option indicating daily incidence of exposure to distressing situations. A limitation here is the use of retrospective ratings when measuring exposure. Thus, two groups were retrospectively defined as ‘not exposed’ and ‘exposed,’ and then “prospectively” compared on outcomes measured at the same time. This type of retrospective cohort design has potential drawbacks due to limited control over potential confounders related to the exposure variable itself. Elaborating on this point, exact recall of past events is often regarded as unreliable. However, in our study we tried not to make claims about the exact prevalence of different difficult situations, but instead used a crude classification of exposure as occurring weekly or less. Moreover, there is a risk that participants who are depressed or in a negative affective state when responding to the questionnaire (i.e., might agree to questions of low job satisfaction or stress of conscience, etc.) may also recall more negative events from the past (i.e., more exposure to difficult situations). Thus, the associations between exposure and outcomes found in this study may, to some degree, have been influenced by a spurious association. Likewise, the bias may also work in the opposite direction, whereby participants in a positive mood when responding to the questionnaire may fail to recall negative events in their retrospective accounts [45]. Additionally, the study included only registered nurses working in Swedish healthcare settings. The results may therefore not be directly transferable to other countries or contexts with different healthcare systems and pandemic responses.

Another methodological consideration concerns the analysis of the qualitative data. To reduce potential bias and enhance the rigor of the analysis of open-ended responses, several strategies were employed. The two researchers (DR and OD), both experienced in qualitative research, independently reviewed and coded the data to minimize individual bias. They engaged in multiple peer debriefing meetings to discuss interpretations, compare codes, and resolve discrepancies. Final consensus was achieved through triangulation with the last author (AR). Throughout the analysis, the researchers maintained a reflexive stance and were continuously aware of how their own preunderstandings could influence the interpretation of the data [51]. To further minimize this influence, all co-authors were invited to review and provide feedback on the analysis, ensuring that multiple perspectives were considered. These steps were undertaken to strengthen the trustworthiness and credibility of the findings.

## Conclusion

The COVID-19 pandemic has had a significant impact on healthcare workers, particularly registered nurses (RNs). Organizational support and leadership play a crucial role in assisting frontline nurses, especially during challenging times such as the COVID-19 pandemic, when they were exposed to distress and ethical dilemmas.

Future strategies should focus on maintaining adequate staffing levels, ensuring competence and teamwork, and providing access to mental health resources and counseling to help nurses manage stress and ethical challenges. Transparent communication is essential to keep nurses informed about changes in protocols and policies. Additionally, flexible scheduling options are necessary to support nurses in balancing their work and personal lives. Implementing these strategies can enable healthcare professionals and systems to respond more effectively to future pandemics and other situations involving increased workloads.

## Data Availability

No datasets were generated or analysed during the current study.
